# Protocol for trapping transient endogenous formaldehyde in live cells to visualize its signaling

**DOI:** 10.1016/j.xpro.2025.104108

**Published:** 2025-09-27

**Authors:** Yuan Pan, Xing-Guang Liang, Xin Li

**Affiliations:** 1 College of Pharmaceutical Sciences, Zhejiang University, Hangzhou 310058, China; 2 First Affiliated Hospital, Zhejiang University School of Medicine, Hangzhou 310003, China; 3 State Key Laboratory of Chinese Medicine Modernization, Innovation Center of Yangtze River Delta, Zhejiang University, Jiaxing 314102, China

**Keywords:** Cell-based Assays, Microscopy, Molecular/Chemical Probes, Biotechnology and bioengineering, Chemistry

## Abstract

Formaldehyde is emerging as a key signaling modulator. Here, we present Trami, a protocol that traps endogenous formaldehyde in live cells and converts its transient signals into stable proximal protein labels for subsequent signaling study. We describe probe design, live-cell administration, and imaging procedures for signaling deconvolution. Trami offers a powerful tool for elucidating formaldehyde-mediated signaling pathways.

For complete details on the use and execution of this protocol, please refer to Pan et al.^1^

## Highlights

• Details on steps for staining cells with the Trami probe
• Procedures for immunofluorescence staining
• Guidance on multiplex imaging
• Instructions for correlating FA levels with proinflammatory signaling

## Abstract (editor highlights)

Publisher’s note: Undertaking any experimental protocol requires adherence to local institutional guidelines for laboratory safety and ethics.

## Graphical abstract

**Figure:**
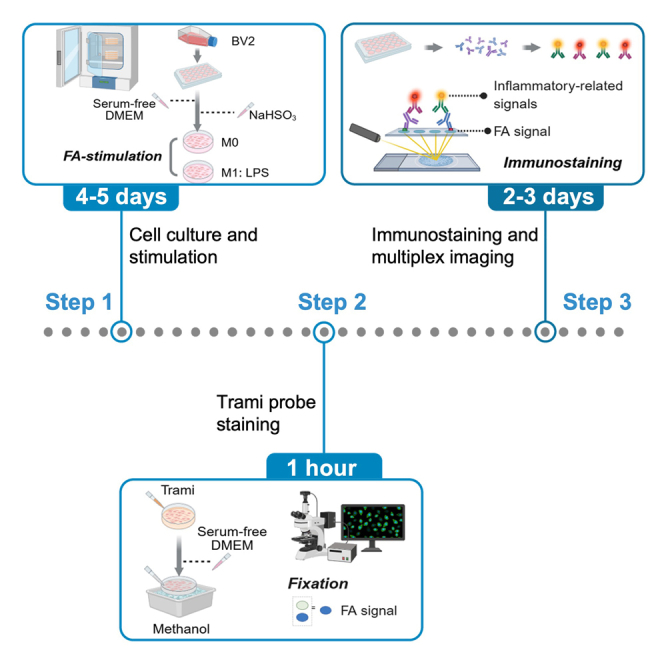

## Before you begin

Metabolites are continuously produced and consumed during cellular metabolism, essential for maintaining physiological function. Among them, reactive metabolites such as formaldehyde (FA), methylglyoxal, *etc*, are critically involved in diverse pathophysiological processes. However, their short half-lives make them difficult to detect. Investigating their signaling mechanisms is even more challenging due to their transient nature.

**Figure 1:**
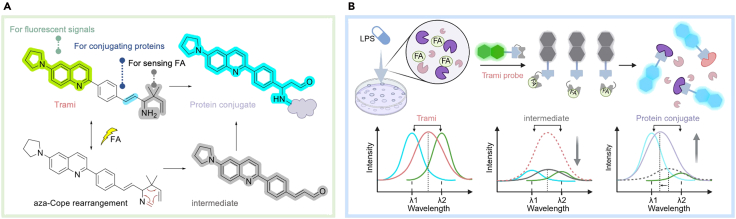
Mechanism and fluorescence response of Trami probe upon FA sensing and subsequently labeling proteins (A) Aza-Cope rearrangement (FA sensing) and protein conjugate formation via covalent labeling, shown via structures. (B) Pipeline scheme of using Trami probe to interrogate FA signaling. Probe’s fluorescence signal change was shown below as cartoon images.

FA is one such reactive metabolite, increasingly recognized for its involvement in various disease-related signaling pathways. To detect endogenous FA and investigate its signaling mechanisms, we developed Trami (trapping for multiplex imaging), a protocol that traps cellular FA and permanently labels its proximal proteins for subsequent imaging-based signaling deconvolution. In this protocol, we outline the detailed procedures for using the Trami method to study FA-associated signaling. The Trami probe consists of a quinoline fluorophore linked *via* an alkenyl spacer to a FA-responsive trigger (a β-gem-dimethyl substituted γ-en-amino group). The probe is cell-membrane permeable and can be administered by incubating live cells in medium containing the dissolved probe. Upon encountering FA in live cells, the trigger undergoes a FA-specific reaction cascade that converts the probe into a protein-reactive intermediate, which covalently labels nearby proteins (Figure 1). In this way, transient FA signals are converted into stable, spatially resolved protein labels. Notably, the FA signal stored by the Trami probe can be visualized both in live and fixed cells by fluorescence imaging, as the probe’s fluorescence signal changes during the sensing cascade: the Trami probe is emissive in both the blue (415-470 nm) and green bands (470-550 nm), the protein-reactive intermediate is almost nonfluorescent, and the probe-protein conjugate is emissive only in the blue band. In this context, an increasing blue/green fluorescence intensity ratio in live cells, or the blue fluorescence intensity alone in fixed cells, can reflect FA levels. When visualized in fixed cells and combined with immunofluorescence staining, Trami enables the correlation of endogenous FA with its associated signaling proteins, supporting high-resolution spatial mapping and mechanistic insights.

This protocol focuses on the staining and imaging workflow using the Trami probe. Detailed synthesis and characterization procedures of the Trami probe have been described in our previous publication.^1^ It is optimized for studying lipopolysaccharide (LPS)-induced FA signaling in the BV2 murine microglial cell line. While applicable to other mammalian cell types and stimuli, optimization of conditions such as probe concentration, incubation time, and staining procedures may be necessary.

### Innovation

Metabolites are critical signaling mediators, but their investigation is often hindered by the lack of reliable detection strategies. Current studies frequently rely on exogenous metabolite addition or manipulation of biosynthetic and catabolic enzymes to infer metabolite functions. Such indirect approaches neglect cellular compensatory mechanisms, meaning endogenous metabolite levels may not reflect experimental predictions. This limitation is particularly severe for reactive and short-lived metabolites like FA, where accurate in situ detection remains challenging. We developed Trami to address this gap. Trami employs a metabolite-activated protein-labeling probe to convert transient FA signals into stable protein tags, enabling simultaneous visualization of FA and its signaling proteins through multiplex immunofluorescence. This chemical-imaging strategy provides spatially resolved and physiologically relevant insight into reactive metabolite signaling, overcoming key limitations of conventional approaches. The protocol offers a step-by-step guide for applying Trami in cultured cells and establishes a broadly applicable platform for probing unstable metabolites in disease-relevant contexts.

### Institutional permissions (if applicable)

All experiments conform to the relevant regulatory standards.

### Design and synthesis of the Trami probe

**Timing: 3–6 months**

1. Design the probe.
  a. Design the FA-specific Trami probe. The probe should contain a fluorophore and a FA-specific reactive group. For the specific design example, see Pan et al.^1^
    i. The probe by itself should be stable and inert towards proteins.
    ii. The probe should show minimum nonspecific binding in live cells and be easily washed out.
    iii. The probe should react specifically with FA and yield a highly reactive intermediate to label proteins.
    iv. Ideally, the probe’s fluorescent signal should change upon reaction with FA and subsequent protein labeling to facilitate interpretation.
2. Synthesize the probe.
  a. Chemically synthesize the probe designed above. For details, see Pan et al.^1^
3. Confirm probe functionality with solution-based assays.
  a. Test the stability of the probe in various biological media by high-performance liquid chromatography (HPLC) and fluorescence spectrometry.
  b. Test the reactivity of the probe towards FA in biologically relevant medium by HPLC and fluorescence spectrometry.
  c. Confirm the specificity of the probe towards FA among various reactive metabolites by HPLC and fluorescence spectrometry.
  d. Test the protein-labeling activity of the probe after activation by FA with bovine serum albumin (BSA) as a model protein *via* matrix-assisted laser desorption/ ionization time-of-flight (MALDI-TOF) mass spectrometry (MS), sodium dodecyl sulfate-polyacrylamide gel electrophoresis (SDS-PAGE), and fluorescence spectrometry.
  e. Decide the optimal excitation and emission wavelengths for the cell-imaging experiments.
  f. Confirm that the final fluorescent signal is dependent on the FA’s dose.

## Key resources table

**Table:** 

REAGENT or RESOURCE	SOURCE	IDENTIFIER
**Antibodies**		

Anti-CD16 antibody (1:400)	Abcam	Cat#ab203883
Goat anti-mouse IgG (H+L)(Elab Fluor 594 conjugated) (1:200)	Elabscience, China	Cat#E-AB-1059
Goat anti-rabbit IgG (H+L)(Elab Fluor 488 conjugated) (1:200)	Elabscience, China	Cat#E-AB-1055
β-tubulin monoclonal antibody (1:2,000)	Elabscience, China	Cat#E-AB-48019

**Chemicals, peptides, and recombinant proteins**		

TBST buffer (10×)	Solarbio, China	Cat#T1081
ProLong glass antifade mountant	Thermo Fisher Scientific	Cat#P36984
Sterile PBS (1×)	Thermo Fisher Scientific	Cat#10010023
Fetal bovine serum (FBS)	Thermo Fisher Scientific	Cat#26170043
Penicillin-streptomycin solution	Thermo Fisher Scientific	Cat#10378016
Dulbecco’s modified Eagle’s medium (DMEM)	Thermo Fisher Scientific	Cat#11995065
0.25% trypsin-EDTA, phenol red (modified)	Thermo Fisher Scientific	Cat#25200056
40% formaldehyde aqueous solution	Sinopharm Chemical, China	Cat#10010018
Sodium bisulfite (NaHSO_3_)	Sigma	Cat#243973-5G
Lipopolysaccharide (LPS)	Meilunbio, China	Cat#MB5198
Methanol	Sinopharm Chemical	Cat#67-56-1
Dimethyl sulfoxide (DMSO)	Beyotime, China	Cat#ST038-500ml
RIPA lysis buffer (medium)	Meilunbio, China	Cat#MA0152
Phenylmethanesulfonyl fluoride (PMSF)	Fudebio-tech, China	Cat#FD0100
Tween 20	Sigma	Cat#P9416
Triton X-100	Sigma	Cat#X100
Bovine serum albumin (BSA)	Sigma	Cat#B2064-10G
Goat serum	Aladdin, China	MP20008-100ml

**Experimental models: Cell lines**		

BV2	Immocell Biotech	Cat#IM-M042

**Software and algorithms**		

Leica S5 software	Leica	N/A
ImageJ	NIH	https://imagej.nih.gov/ij/download.html

**Other**		

Confocal dish (20 mm)	Nest	N/A
Cell culture plates	Corning	N/A
Cell cryovials	Corning	N/A
T25 or T75 cell culture flasks	Corning	N/A
15 or 50 mL centrifuge tubes	Corning	N/A
Cell crawling slides	WHB Scientific	N/A
Refrigerated centrifuge	Thermo Scientific	N/A
4°C centrifuge	Thermo Scientific	N/A
Double distillation water purification instrument	Millipore	N/A
Standard optical microscope	Olympus	N/A
Pipettes	Eppendorf	N/A
Biological safety cabinet	Thermo Scientific	N/A
CO_2_ incubator	Thermo Scientific	N/A
37°C thermostatic water bath	Thermo Scientific	N/A
4°C and −20°C freezers	Midea, China	N/A
Ice machine	Scotsman, Italy	N/A
Inverted laser confocal microscope (FV1000)	Olympus	N/A
Confocal laser scanning microscope TCS SP8 Leica Microsystems N/A	Leica	N/A

## Materials and equipment

**Table:** Complete DMEM medium

Reagent	Final concentration	Volume (mL)
DMEM	89%	44.5
FBS	10%	5
Penicillin-streptomycin solution (100 U/mL)	1%	0.5
**Total**	**100%**	**50**

**Table:** Serum-free DMEM medium

Reagent	Final concentration	Volume (mL)
DMEM	99%	49.5
Penicillin-streptomycin solution	1%	0.5
**Total**	**100%**	**50**

***Note:*** All media are stored at 4°C up to 2 months.

### Reagents for Trami probe staining

• Trami probe stock solution: Dissolve an appropriate amount of Trami probe with DMSO to 5 mM.
• Trami probe working solution: Dilute the stock solution with serum-free DMEM medium to 5 μM.

***Note:*** The probe stock solution is stored at −20°C for short-term storage up to 6 months. Avoid repeat freeze/thaw cycles. The working solution should be prepared fresh during the procedure. For inexperienced experimenters, an additional critical detail is to store the stock solution in the dark at all times (e.g., using amber glass vials or wrapping containers in aluminum foil) to prevent light-induced degradation, as many probes are photosensitive. When preparing working solutions, these must be made fresh immediately before use during the experimental procedure. Ensure working solutions are also protected from light until they are added to samples, to preserve their integrity.

## Step-by-step method details

### Cell propagation

**Timing: 3–4 days**

This section describes cell preparation for the study.

1. Cell thawing, seeding, and culture.
  a. Retrieve cryopreserved BV2 microglia (1–5 × 10^6^ cells/vial in 1 mL serum-free cryopreservation medium) from liquid nitrogen storage.
    i. Thaw rapidly in a pre-set 37°C water bath (1–2 min).
    ii. Swirl gently until only a small ice crystal remains.
  b. Transfer thawed cells into a 15-mL conical tube containing 10 mL pre-warmed complete DMEM medium.
    i. Mix gently by inversion.
    ii. Centrifuge at 1000 rpm (200 × g) for 5 min at 25°C.
    iii. Discard the supernatant.
    iv. Resuspend the pellet in 2 mL fresh complete medium.
  c. Measure cell concentration, then dilute cell suspension to 1.5×10^5^ viable cells/mL using fresh complete DMEM medium.
  d. Seed 5 mL of cell suspension into a T25 cell culture flask.
  e. Incubate at 37°C, 5% CO_2_ in a humidified incubator until the cells reach 80%–90% confluence (typically 2–3 days).
  f. Discard the spent culture medium.
    i. Add 1 mL of pre-warmed sterile PBS.
    ii. Gently agitate the flask twice to rinse off residual complete medium.
    iii. Introduce 500 μL of 0.25% Trypsin-EDTA solution.
    iv. Incubate the flask at 37°C for 2 min.
  g. Monitor cells under an inverted optical microscope.
    i. Upon observing cell rounding, terminate digestion by adding DMEM complete medium supplemented with 10% FBS.
    ii. Use a pipette to gently triturate the flask bottom to detach cells.
    iii. Transfer the cell suspension to a 5-mL Eppendorf (EP) tube.
    iv. Seal with parafilm.
    v. Centrifuge at 1000 rpm for 5 min.
    vi. Discard the supernatant.
    vii. Resuspend the cell pellet in fresh complete medium via gentle pipetting until homogeneous.
  h. Cell seeding.
    i. Place poly-L-lysine (0.1% (w/v) in sterile distilled water or PBS, 30–60 min)-pretreated cell culture slides in a 24-well plate.
    ii. Transfer the cell suspension to the wells at a seeding density of 2×10^5^ cells per well.
    iii. Allow cells to adhere for a minimum of 24 hours before subsequent experiments.

**CRITICAL:** Cells with passages 5–8 were used.

### Stimulating cells with lipopolysaccharide

**Timing: 1 day**

**Figure 2:**
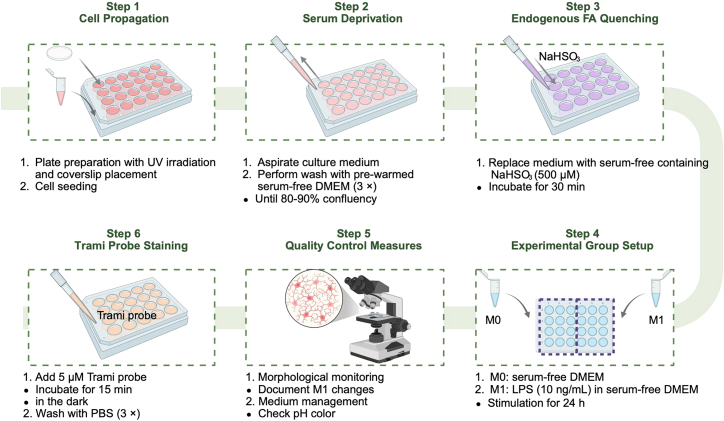
Procedures for cell modeling and staining with Trami probe

This section describes the establishment of M0/M1-polarized BV2 microglia for correlating endogenous FA levels with proinflammatory signaling (Figure 2).

2. Serum deprivation.
  a. Aspirate culture medium gently once the cells reach the target confluence.
  b. Perform three sequential washes with pre-warmed serum-free DMEM (37°C).

**CRITICAL:** This step is essential to remove residual serum, ensuring that the Trami probe’s fluorescence labels endogenous proteins in response to formaldehyde.

3. Basal FA depletion.
  a. Incubate cells with serum-free DMEM containing 500 μM NaHSO_3_ in a 37°C, 5% CO_2_ incubator for 30 min to eliminate endogenous basal FA within the cells.
  b. Perform three sequential washes with preheated sterile PBS (pH 7.4, 37°C).

**CRITICAL:** This step is essential to eliminate basal signals from endogenous aldehydes, ensuring specific detection of LPS-induced FA by the Trami probe.

4. LPS-stimulation.
  a. Treatment allocation.
    i. Control group (M0): Add 1 mL serum-free DMEM per well.
    ii. LPS group (M1): Prepare 10 ng/mL LPS solution in serum-free DMEM. Add 1 mL per well (final LPS concentration: 10 ng/mL).
  b. Stimulation period: Incubate both groups at standard culture conditions (37°C, 5% CO_2_) for 24 h.
5. Quality control measure.
  a. Morphological monitoring: Examine cells every 6 - 8 h using phase-contrast microscopy. Document M1 phenotype changes (amoeboid shape, membrane ruffling) in the LPS-treated group versus spindle-shaped morphology in controls.
  b. Medium management: Check pH indicator color every 12 h.

Notes: If the color turns yellow (pH < 7.0), replace half with fresh preheated medium to keep optimal conditions. For the M1 polarization group treated with LPS, ensure the fresh medium added during this replacement is supplemented with LPS at the same concentration used for initial stimulation. Use sterile technique to avoid contamination during medium exchange.

**CRITICAL:** This step is critical to maintain a consistent LPS concentration throughout the incubation period, as dilution from medium replacement could reduce the stimulus intensity and compromise M1 polarization efficiency.

### Trami probe staining

**Timing: 1 h**

This section describes probe staining procedures (Figure 2).

6. Probe staining.
  a. Aspirate culture medium and perform three sequential washes with pre-warmed serum-free DMEM (37°C) to remove LPS.
  b. Prepare 5 μM Trami probe in serum-free DMEM.
  c. Add 1 mL/well to cell monolayers and incubate at 37°C for 15 min in the dark.
  d. Aspirate probe-containing medium and perform three rapid washes with preheated PBS (pH 7.4).
  e. Add 1 mL/well of serum-free DMEM, and allow the probe to respond and label formaldehyde for 1 hour in an atmosphere of 5% CO_2_ at 37°C.

***Note:*** Protect from light to prevent photobleaching of the fluorogenic probe.

**CRITICAL:** Residual unbound probe may contribute to nonspecific background fluorescence. Ensure thorough washing to enhance the signal-to-noise ratio.

### Cell fixation

**Timing: 0.5 h**

**Figure 3:**
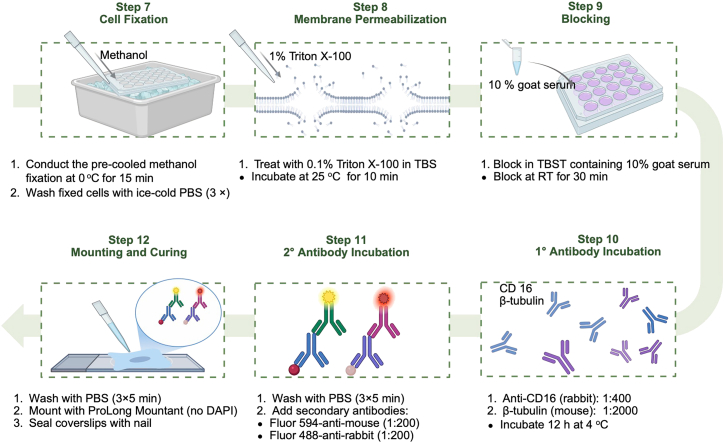
Procedures for staining cells with antibodies and cell mounting for multiplex imaging

This section is to fix cells for immunostaining (Figure 3).

7. Cell fixation.
  a. Replace medium with 2 mL pre-cooled (−20°C) methanol.
  b. Fix cells on an ice bath for 15 min.
  c. Wash fixed cells 3 times with ice-cold PBS (5 min per wash).

***Purpose:*** To preserve the cell structure and remove the residual probe in the cells.

### Immunostaining

**Timing: 2 days**

This section describes immuno-staining procedures. The protein marker used here is CD16, with β-tubulin used as a control (Figure 3).

8. Membrane permeabilization.
  a. Treat the cells with 0.1% Triton X - 100 in TBS (1 mL/well).
  b. Incubate at 25°C for 10 min.

***Purpose:*** To create pores in the cell membrane, allowing antibodies to access the target proteins inside the cells.

9. Blocking.
  a. Incubate the cells in TBST containing 10% goat serum + 0.1% Triton X-100 (1 mL/well).
  b. Block at ambient temperature for 30 min.

***Purpose:*** To reduce non-specific binding of antibodies to the cell surface, minimizing background noise in subsequent steps.

10. Primary antibody incubation.
  a. Dilute antibodies in blocking buffer:
    i. Anti - CD16 (rabbit monoclonal): 1:400.
    ii. β-tubulin (mouse monoclonal): 1:2000.
  b. Add 500 μL/well and incubate 12 h in a 4°C freezer.

***Purpose:*** To enable the primary antibodies to specifically bind to their target proteins.

11. Secondary antibody incubation.
  a. Wash cells 3× with ice-cold PBS (5 min per wash at ambient temperature).
  b. Add fluorescently conjugated secondary antibodies:
    i. Fluor 594 - anti - mouse IgG: 1:200 in blocking buffer (3% BSA in PBS with 0.1% Tween-20 (for β-tubulin, red channel).
    ii. Fluor 488 - anti - rabbit IgG: 1:200 in the same blocking buffer (for CD16, green channel).
  c. Incubate at ambient temperature for 1 h in the dark.

***Purpose:*** To visualize primary antibody binding via fluorescence; ensure spectral separation from Trami probe (e.g., Trami: blue, CD16: green, β-tubulin: red).

***Note:*** Protect from light to prevent photobleaching.

12. Mounting and Curing.
  a. Wash cells 3 × with PBS (5 min per wash at ambient temperature).
  b. Mount with ProLong Gold Antifade Mountant (non-fluorescent formula).
  c. Seal coverslips with nail polish and cure at ambient temperature for 24 h in the dark.

***Purpose:*** To prepare the cells for imaging under a fluorescence microscope, ensuring proper sample preservation and optimal visualization.

***Note:*** Store slides at 4°C protected from light; use within 1 week for best signal retention.

### Multiplex imaging

**Timing: 2–6 h**

**Figure 4:**
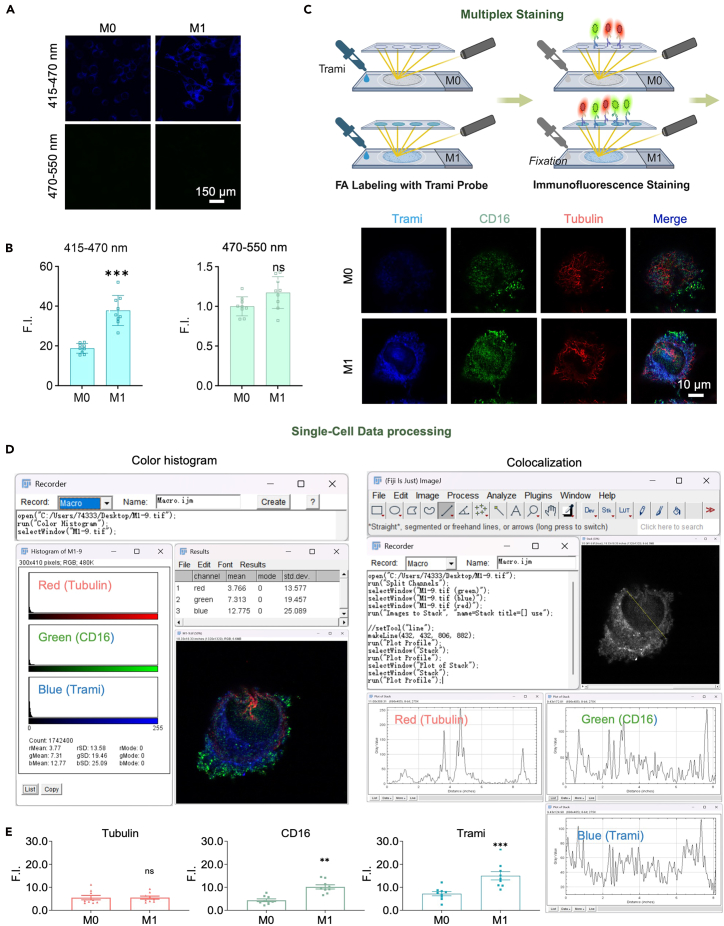
Multiplex imaging and single-cell analysis of BV2 cells after LPS stimulation and Trami probe staining (A) Fluorescent images of BV2 cells upon M0 (serum-free DMEM) or M1 (LPS-treated) modeling cells, followed by Trami probe staining and fixation. (B) Bar graphs showing fluorescence intensity (F. I.) of M0/M1 cells at the indicated wavelength ranges. (C) Scheme of multiplex staining and imaging workflow (Trami labeling, immunofluorescence for CD16/Tubulin) and representative images of M0/M1 cells after multiplex imaging. (D) Single-cell data processing: color histogram analysis of Tubulin (red), CD16 (green), Trami (blue); and colocalization analysis with line scan profiles. (E) Quantification of F.I. for Tubulin, CD16, Trami in M0/M1 cells. Results are shown as mean ± sd, n = 9. The student’s t-test is used to compare the means between two groups. ns, no significant difference; ∗∗ p<0.01; ∗∗∗p<0.001.

This section describes the multiplex imaging strategy for data acquisition (Figure 4).

13. Confocal microscope setup.
  a. Instrument: Leica STELLARIS 5 full-spectrum laser confocal microscope with HyD hybrid detectors and spectral unmixing.
  b. Objective: 63 × HC PL APO oil-immersion (NA 1.4).
  c. Software: LAS X with Lightning super-resolution module.
14. Acquisition settings.
  a. Blue channel (Trami): λ_ex_ = 405 nm, λ_em_ = 415–470 nm.
  b. Green channel (CD16): λ_ex_ = 495 nm, λ_em_ = 500–555 nm.
  c. Red channel (β-tubulin): λ_ex_ = 590 nm, λ_em_ = 600–640 nm.
15. Imaging Workflow.
  a. Capture single-cell images in super-resolution mode and save them as 1024×1024, 12-bit TIFF images.
  b. Acquire Z-stack images (0.5 μm step size) covering the entire cell volume.
  c. Capture 9–12 random fields of view (FOVs) per dish, n≥3 independent experiments.
  d. Save as TIFF files (no compression) with metadata (e.g., date, exposure settings).

***Note:*** Randomly select multiple fields of view for imaging, and repeat the imaging process for at least three independent experiments.

16. Data visualization and statistics.
  a. Software: GraphPad Prism 10.
  b. Plots:
    i. Heatmaps: Co-localization intensity matrices (FA vs. CD16).
    ii. Line graphs: Mean ± SEM of co-localization coefficients across time points.

**Table:** 

Data analysis	Operation content	Key Parameters / Equipment	Operation purpose
Color Histogram Analysis	1. Analyze the merged RGB images	Color Histogram tool	Evaluate fluorescence intensity of CD16, β-tubulin, and Trami channels
	2. Standardize workflow	Plugins > Macros > Record (optional)	Improve experiment reproducibility
Colocalization Analysis	1. Separate and align channels	Image > Color > Split Channels; Image > Stacks > Images to Stack	Prepare for colocalization analysis
	2. Quantify line profiles	Analyze > Plot Profile X-axis: Pixel positions Y-axis: Grayscale intensity (0-255)	Analyze fluorescence intensity distribution

***Note:*** Perform at least three independent experiments for statistical significance. Maintain consistent microscopy settings for all imaging sessions.

## Expected outcomes

Using the above methods, LPS-treated cells (M1) will exhibit higher Trami probe fluorescence intensity in the 415-470 nm range after fixation compared to control cells (M0) (Figures 4A and 4B). Both groups should show minimal green band fluorescence (470-550 nm), indicating effective washout of unreacted probe. The immunofluorescence signal of CD16 in the M1 group will be stronger than that in the M0 group; while β-tubulin staining should remain comparable between groups (Figure 4E). Notably, the fold increase of Trami fluorescence intensity in M1 versus M0 closely mirrors that of the CD16 signal, suggesting a parallel upregulation. Moderate spatial co-localization between Trami and CD16 signals is expected upon analysis, supporting a potential role for endogenous formaldehyde in proinflammatory signaling.

## Limitations

While Trami enables the visualization of endogenous formaldehyde (FA) signaling with high spatial resolution, several limitations should be noted. First, Trami captures the intensity and spatial correlation between FA and its associated signaling proteins but does not directly reveal the underlying molecular mechanisms. Additional biochemical or genetic experiments are required to validate the functional roles of candidate proteins. Second, Trami does not identify the specific proteins labeled by the activated probe. While the protein labeling reflects proximity to FA activity, further analysis, such as affinity enrichment and mass spectrometry, is needed to determine protein identities. Third, as an imaging-based method, Trami relies on conventional fluorescence microscopy and is constrained by spectral overlap, which limits its ability to profile multiple signaling nodes in parallel. Fourth, Trami converts transient FA signals into stable protein labels, capturing cumulative exposure rather than real-time dynamics. This may obscure short-lived or reversible signaling events.

## Troubleshooting

### Problem 1

Weak Trami probe fluorescence (related to Trami probe staining).

### Potential solution

This may occur when the Trami probe is decomposed or when cellular FA levels are very low. For the former, check the stability of the probe stock. For the latter, a positive control experiment, where cells are fed with exogenous FA, can be employed.

### Problem 2

The Trami probe emits fluorescence in the green band (related to cell fixation).

### Potential solution

This may happen when the washout procedure is inadequate and the unreacted probe remains in the cells. If a clear green fluorescence signal from the Trami probe is detected, attempt to wash the cells three more times.

### Problem 3

Excessive photobleaching of fluorescent signals (related to multiplex imaging).

### Potential solution

This could result from prolonged exposure to excitation light. Reduce the excitation light intensity and shorten exposure times during imaging to minimize photobleaching. Additionally, select fluorophores with higher photostability (e.g., Alexa Fluor dyes) instead of less stable ones (e.g., FITC) for the second antibody. Using anti-fade mounting media can also help preserve fluorescence signals by reducing reactive oxygen species generated during excitation.

### Problem 4

Nonspecific antibody labeling (related to immunostaining).

### Potential solution

This may occur due to insufficient blocking or incompatible antibody combinations. For insufficient blocking, select high-quality fetal bovine serum as a blocking solution. When co-incubating two primary antibodies, ensure their sources are different, and the fluorescent secondary antibodies correspond to them with non-overlapping fluorescence bands.

### Problem 5

Poor cell morphology on coverslips (related to Cell seeding).

### Potential solution

This may happen due to improper coverslip coating or suboptimal cell seeding density. For coverslip coating, use appropriate substances like poly - L - lysine or fibronectin to enhance cell adhesion. For cell seeding, adjust the density to ensure cells are neither too sparse nor too crowded; a moderate density allows cells to spread well and maintain normal morphology. Also, check the cell culture medium for freshness and proper composition to support healthy cell growth.

## Resource availability

### Lead contact

Further information and requests for resources and reagents should be directed to and will be fulfilled by the lead contact, Xin Li (lixin81@zju.edu.cn).

### Technical contact

Technical questions on executing this protocol should be directed to and will be answered by the technical contact, Yuan Pan (panyuan1230123@zju.edu.cn).

### Materials availability

The Trami probe generated in this study is available from the lead contact for research purposes, depending on stock availability.

### Data and code availability

The previously published article partly includes the data shown in this protocol. Any additional information for reanalyzing the data reported in this paper is available from the lead contact upon request.

## Acknowledgments

This work was supported by the National Natural Science Foundation of China (22377106) and the Natural Science Foundation of Zhejiang Province (LZ23H300001). We appreciate Chen Liu from the microscopy platform, Central Laboratory, the First Affiliated Hospital, Zhejiang University School of Medicine, for technical support. The graphical abstract/figures were created using Biorender.com.

## Author contributions

Conceptualization, X.L.; methodology, Y.P.; investigation, Y.P.; writing, Y.P. and X.L.; supervision, X.-G.L. and X.L.

## Declaration of interests

The authors declare no competing interests.

## Declaration of generative AI and AI-assisted technologies in the writing process

During the preparation of this work, the authors used ChatGTP in order to revise the grammar and polish the language. After using this tool/service, the authors reviewed and edited the content as needed and take full responsibility for the content of the publication.
